# Supplementary material for: Hybrid Models and Biological Model Reduction with PyDSTool
Source: PLoS Comput Biol. 2012 Aug 9;8(8):e1002628. doi: 10.1371/journal.pcbi.1002628 (PMC3415397; doi:10.1371/journal.pcbi.1002628)
Supplement: Text S4 — Complete source code for the PyDSTool package (version 0.88.120504). Includes API documentation and help files linking to web pages. This file is identical to the current public release on Sourceforge.net. (ZIP) [file pcbi.1002628.s004.zip › PyDSTool/html/PyDSTool.Toolbox.neuro_data.get_burst_spikes-class.html]

xml version="1.0" encoding="ascii"?


PyDSTool.Toolbox.neuro\_data.get\_burst\_spikes


| Home | Trees | Indices | Help | PyDSTool |
| --- | --- | --- | --- | --- |

Package PyDSTool ::
Package Toolbox ::
Module neuro\_data ::
Class get\_burst\_spikes

[hide private]

[frames] | no frames]

# Class get\_burst\_spikes

source code

```
       object --+            
                |            
 MProject.feature --+        
                    |        
MProject.feature_node --+    
                        |    
 MProject.ql_feature_node --+
                            |
                           get_burst_spikes
```

---

Requires a get\_spike\_data and get\_spike\_model instance to be the only
sub-features (supplied as a dict with keys 'is\_spike\_data' and
'is\_spike\_model').


|  |  |
| --- | --- |
| Instance Methods | [hide private] |

|  |  |
| --- | --- |
| \_local\_init(self) | source code |

|  |  |
| --- | --- |
| postprocess\_ref\_traj(self)  User-definable by overriding in a sub-class | source code |

|  |  |
| --- | --- |
| evaluate(self, target)  Default method: evaluate sub-features in order (assumes they are stored as a list). | source code |

|  |  |
| --- | --- |
| \_eval(self, traj, burst\_est, is\_spike) | source code |

**Inherited from `MProject.feature_node`**:
`__getitem__`,
`__init__`,
`__repr__`,
`__str__`,
`propagate_verbosity`,
`set_ref_traj`

**Inherited from `MProject.feature_node`** (private):
`_residual_info`

**Inherited from `MProject.feature`**:
`__call__`,
`__eq__`,
`__hash__`,
`__ne__`,
`finish`,
`info`,
`prepare`,
`reset_metric`,
`validate`

**Inherited from `MProject.feature`** (private):
`_find_idx`

**Inherited from `object`**:
`__delattr__`,
`__getattribute__`,
`__new__`,
`__reduce__`,
`__reduce_ex__`,
`__setattr__`


|  |  |
| --- | --- |
| Properties | [hide private] |

**Inherited from `object`**:
`__class__`


|  |  |
| --- | --- |
| Method Details | [hide private] |

|  |  |
| --- | --- |
| postprocess\_ref\_traj(self) | source code |

User-definable by overriding in a sub-class

Overrides: MProject.feature.postprocess\_ref\_traj: *(inherited documentation)*

|  |  |
| --- | --- |
| evaluate(self, target) | source code |

Default method: evaluate sub-features in order (assumes they are
stored as a list).

Can override with more sophisticated method (e.g. for use with a
dictionary of sub-features).

Overrides: MProject.feature.evaluate

  


| Home | Trees | Indices | Help | PyDSTool |
| --- | --- | --- | --- | --- |

|  |  |
| --- | --- |
| Generated by Epydoc 3.0.1 on Fri May 4 15:24:09 2012 | http://epydoc.sourceforge.net |
